# Bis(ethanol-κ*O*)bis­(pyridine-3-carb­aldehyde-κ*N* thio­semicarbazone)bis­(thio­cyanato-κ*N*)iron(II)–pyridine-3-carbaldehyde thio­semicarbazone (1/2)

**DOI:** 10.1107/S1600536809024076

**Published:** 2009-07-01

**Authors:** Shao-Mei Wang

**Affiliations:** a College of Chemistry and Chemical Engineering, Anyang Normal University, Anyang, Henan 455000, People’s Republic of China

## Abstract

The crystal structure of the title Fe^II^ complex, [Fe(NCS)_2_(C_7_H_8_N_4_S)_2_(CH_3_CH_2_OH)_2_]·2C_7_H_8_N_4_S, based on the Schiff base ligand pyridine-3-carbaldehyde thio­semicarbazone (pct), results from the cocrystallization of an Fe^II^ coordination compound together with two of the pct ligands. The complex unit is mononuclear, with the central Fe^II^ ion located on a crystallographic centre of inversion and coordinated by four N atoms from two pct ligands and two thio­cyanate anions. The slightly distorted octa­hedral coordination is completed by two O atoms from ethanol mol­ecules. The crystal packing is accomplished inter­molecular N—H⋯S hydrogen bonds.

## Related literature

For the structures of metal complexes of Schiff base ligands synthesized by condensation of pyridine-3-carbaldehyde and amino compounds, see: Brook *et al.* (2000 ▶); Deng *et al.* (2007 ▶); Garbelini *et al.* (2008 ▶); Kowol *et al.* (2007 ▶); Zhong *et al.* (2007 ▶). For the corresponding Mn(II) complex of pyridine-3-carbaldehyde thio­semicarbazone, see: Li *et al.* (2006 ▶).
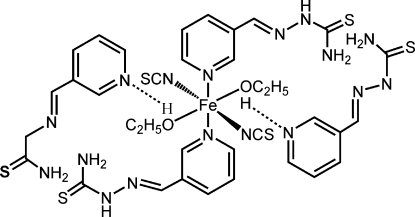


## Experimental

### 

#### Crystal data

[Fe(NCS)_2_(C_7_H_8_N_4_S)_2_(C_2_H_6_O)_2_]·2C_7_H_8_N_4_S
*M* *_r_* = 985.08
Triclinic, 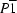

*a* = 8.916 (4) Å
*b* = 9.556 (5) Å
*c* = 14.538 (7) Å
α = 87.341 (8)°
β = 88.191 (8)°
γ = 69.604 (8)°
*V* = 1160 (1) Å^3^
*Z* = 1
Mo *K*α radiation
μ = 0.65 mm^−1^
*T* = 173 K
0.32 × 0.26 × 0.22 mm

#### Data collection

Bruker SMART CCD area-detector diffractometer
Absorption correction: multi-scan (*SADABS*; Sheldrick, 1996 ▶) *T*_min_ = 0.819, *T*_max_ = 0.871
8300 measured reflections
4037 independent reflections
2953 reflections with *I* > 2σ(*I*)
*R*_int_ = 0.022

#### Refinement

*R*[*F* ^2^ > 2σ(*F* ^2^)] = 0.042
*wR*(*F* ^2^) = 0.134
*S* = 1.01
4037 reflections
282 parameters
1 restraint
H atoms treated by a mixture of independent and constrained refinement
Δρ_max_ = 0.43 e Å^−3^
Δρ_min_ = −0.24 e Å^−3^

### 

Data collection: *SMART* (Bruker, 1998 ▶); cell refinement: *SAINT* (Bruker, 1998 ▶); data reduction: *SAINT*; program(s) used to solve structure: *SHELXS97* (Sheldrick, 2008 ▶); program(s) used to refine structure: *SHELXL97* (Sheldrick, 2008 ▶); molecular graphics: *SHELXTL* (Sheldrick, 2008 ▶); software used to prepare material for publication: *SHELXTL*.

## Supplementary Material

Crystal structure: contains datablocks I, global. DOI: 10.1107/S1600536809024076/im2122sup1.cif

Structure factors: contains datablocks I. DOI: 10.1107/S1600536809024076/im2122Isup2.hkl

Additional supplementary materials:  crystallographic information; 3D view; checkCIF report

## Figures and Tables

**Table 1:** Hydrogen-bond geometry (Å, °)

*D*—H⋯*A*	*D*—H	H⋯*A*	*D*⋯*A*	*D*—H⋯*A*
N9—H9*B*⋯S3^i^	0.88	2.67	3.533 (3)	165
N4—H4*B*⋯S1^ii^	0.88	2.52	3.373 (3)	164
N4—H4*A*⋯S2	0.88	2.80	3.360 (3)	123
N3—H3⋯S3^iii^	0.88	2.59	3.414 (3)	156

Symmetry codes: (i) 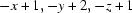; (ii) 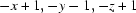; (iii) 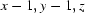.

**Fig. 1.**
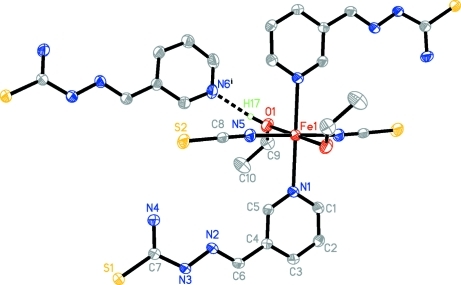
The structure of (I) × pct, symmetry code: (i) -x + 2, -y, -z + 1.

**Fig. 2.**
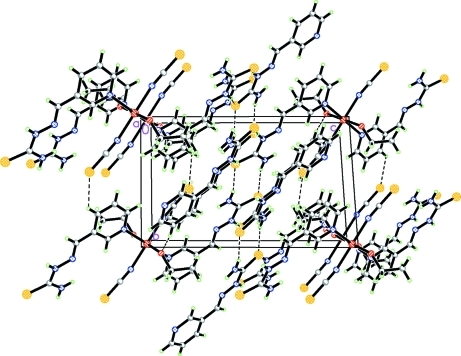
Three-dimensional supramolecular network constructed by hydrogen bonding interactions (dashed lines).

## supplementary crystallographic information

### Comment

Several crystal structures of metal complexes based on Schiff base ligands being synthesized by condensation of pyridine-3-carbaldehyde and amino compounds have been previously reported (Brook *et al.*, 2000; Deng *et al.*, 2007; Garbelini *et al.*, 2008; Kowol *et al.*, 2007; Zhong *et al.*, 2007). However, with regard to pyridine-3-carbaldehyde thiosemicarbazone (pct), only the corresponding Mn(II) complex has been documented (Li *et al.*, 2006). Herein, we report the synthesis and crystal crystal structure of its Fe^II^ complex.

The structure of the title compound, (I), consists of discrete [Fe(C_7_H_8_N_4_S)_2_(SCN)_2_(C_2_H_5_OH)_2_] neutral units and uncoordinated pct molecules. The two semicarbazone ligands are planar, and each binds to Fe^II^ via the pyridine N atom. Therefore, pct acts as a monodentate ligand with the sulphur and nitrogen atoms of the semicarbazone subunit remaining uncoordinated. In addition to pct, the central Fe^II^ is coordinated by two SCN^-^ anions and two C_2_H_5_OH molecules via the N and O atoms, respectively. (Fig. 1)

The molecules are held together by intermolecular hydrogen bonding interactions. The imino nitrogen N3 acts as a hydrogen donor site towards S3 of the uncoordinated pct molecule forming intermolecular N3—H3···S3 hydrogen bonds. S3 also accepts a hydrogen bond from N9 of a neighboring uncoordinated pct to form N9—H9B···S3 hydrogen bonds. In addition, the SCN^-^ also is involved in the construction of the hydrogen bond network by establishing an interaction between the terminal S atoms accept and the hydrogen atom at N4 to form N4—H4A···S2 and N4—H4B···S1 hydrogen bonds (Table 1, Fig. 2).

### Experimental

A mixture of 0.5 mmol FeCl_2_ × 6 H_2_O, 1.0 mmol of (NH_4_)SCN and 10 ml water-ethanol (1:2 *v*/*v*) was stirred for *ca.* 2 hrs at 343 K. Then 1.0 mmol pct in 10 ml water-ethanol mixture (1:2 *v*/*v*) was added. The mixture was further stirred for another 2 h, then filtered. The resultant filtrate was left to stand for slow evaporation at room temperature. Dark green single crystals of (I) suitable for X-ray structure analysis were obtained after a period of one week (yield 63%).

### Refinement

Hydrogen atoms attached to carbon atoms and nitrogen atoms were positioned geometrically and treated as riding, with C—H = 0.93 Å, N—H = 0.86 Å, and *U*_iso_(H) = 1.2*U*_eq_(C or N). The H atom attached to the hydroxy group of the ethanol ligand was located from difference density maps and was refined with distance restraints of O–H = 0.82 (1) Å.

### Crystal data

**Table:**

[Fe(NCS)_2_(C_7_H_8_N_4_S)_2_(C_2_H_6_O)_2_]·2C_7_H_8_N_4_S	*Z* = 1
*M**_r_* = 985.08	*F*(000) = 512
Triclinic, *P*1	*D*_x_ = 1.411 Mg m^−^^3^
Hall symbol: -P 1	Mo *K*α radiation, λ = 0.71073 Å
*a* = 8.916 (4) Å	Cell parameters from 3773 reflections
*b* = 9.556 (5) Å	θ = 2.3–26.9°
*c* = 14.538 (7) Å	µ = 0.65 mm^−^^1^
α = 87.341 (8)°	*T* = 173 K
β = 88.191 (8)°	Block, dark green
γ = 69.604 (8)°	0.32 × 0.26 × 0.22 mm
*V* = 1160 (1) Å^3^	

### Data collection

**Table:**

Bruker SMART CCD area-detector diffractometer	4037 independent reflections
Radiation source: fine-focus sealed tube	2953 reflections with *I* > 2σ(*I*)
graphite	*R*_int_ = 0.022
φ and ω scans	θ_max_ = 25.0°, θ_min_ = 1.4°
Absorption correction: multi-scan (*SADABS*; Sheldrick, 1996)	*h* = −10→10
*T*_min_ = 0.819, *T*_max_ = 0.871	*k* = −11→11
8300 measured reflections	*l* = −17→17

### Refinement

**Table:**

Refinement on *F*^2^	Primary atom site location: structure-invariant direct methods
Least-squares matrix: full	Secondary atom site location: difference Fourier map
*R*[*F*^2^ > 2σ(*F*^2^)] = 0.042	Hydrogen site location: inferred from neighbouring sites
*wR*(*F*^2^) = 0.134	H atoms treated by a mixture of independent and constrained refinement
*S* = 1.01	*w* = 1/[σ^2^(*F*_o_^2^) + (0.082*P*)^2^ + 0.3403*P*] where *P* = (*F*_o_^2^ + 2*F*_c_^2^)/3
4037 reflections	(Δ/σ)_max_ = 0.001
282 parameters	Δρ_max_ = 0.43 e Å^−^^3^
1 restraint	Δρ_min_ = −0.24 e Å^−^^3^

### Special details

**Table:**

Geometry. All e.s.d.'s (except the e.s.d. in the dihedral angle between two l.s. planes) are estimated using the full covariance matrix. The cell e.s.d.'s are taken into account individually in the estimation of e.s.d.'s in distances, angles and torsion angles; correlations between e.s.d.'s in cell parameters are only used when they are defined by crystal symmetry. An approximate (isotropic) treatment of cell e.s.d.'s is used for estimating e.s.d.'s involving l.s. planes.
Refinement. Refinement of *F*^2^ against ALL reflections. The weighted *R*-factor *wR* and goodness of fit *S* are based on *F*^2^, conventional *R*-factors *R* are based on *F*, with *F* set to zero for negative *F*^2^. The threshold expression of *F*^2^ > σ(*F*^2^) is used only for calculating *R*-factors(gt) *etc*. and is not relevant to the choice of reflections for refinement. *R*-factors based on *F*^2^ are statistically about twice as large as those based on *F*, and *R*- factors based on ALL data will be even larger.

### Fractional atomic coordinates and isotropic or equivalent isotropic displacement parameters (Å^2^)

**Table:**

	*x*	*y*	*z*	*U*_iso_*/*U*_eq_	
Fe1	0.5000	0.0000	0.0000	0.0416 (2)	
N1	0.2848 (3)	0.1196 (3)	0.09806 (16)	0.0410 (6)	
N2	0.1975 (3)	−0.0995 (3)	0.33648 (15)	0.0389 (6)	
N3	0.1640 (3)	−0.1698 (3)	0.41443 (16)	0.0413 (6)	
H3	0.0676	−0.1382	0.4397	0.050*	
N4	0.4186 (3)	−0.3344 (3)	0.40767 (19)	0.0659 (9)	
H4A	0.4316	−0.2885	0.3557	0.079*	
H4B	0.4981	−0.4112	0.4298	0.079*	
N5	0.5606 (3)	−0.2022 (3)	0.08302 (19)	0.0536 (7)	
C12	0.7900 (4)	0.3134 (4)	0.8792 (2)	0.0604 (9)	
H12	0.6944	0.3331	0.9150	0.072*	
N7	0.8316 (3)	0.5754 (3)	0.64455 (16)	0.0424 (6)	
N8	0.8582 (3)	0.6617 (3)	0.57213 (17)	0.0453 (6)	
H8	0.9546	0.6403	0.5474	0.054*	
N9	0.5924 (3)	0.7961 (3)	0.5738 (2)	0.0652 (8)	
H9A	0.5814	0.7316	0.6163	0.078*	
H9B	0.5080	0.8711	0.5547	0.078*	
O1	0.6741 (3)	0.0634 (3)	0.07601 (15)	0.0541 (6)	
S1	0.24701 (9)	−0.36692 (9)	0.55347 (5)	0.0500 (2)	
S2	0.64184 (10)	−0.43494 (10)	0.21504 (7)	0.0632 (3)	
S3	0.76674 (9)	0.89666 (9)	0.45668 (6)	0.0525 (3)	
C1	0.1770 (4)	0.2554 (3)	0.0754 (2)	0.0457 (7)	
H1	0.1962	0.3076	0.0218	0.055*	
C2	0.0403 (4)	0.3210 (3)	0.1271 (2)	0.0471 (7)	
H2	−0.0332	0.4165	0.1092	0.057*	
C3	0.0117 (3)	0.2462 (3)	0.20499 (19)	0.0416 (7)	
H3A	−0.0815	0.2902	0.2418	0.050*	
C4	0.1196 (3)	0.1064 (3)	0.22943 (18)	0.0354 (6)	
C5	0.2540 (3)	0.0486 (3)	0.17329 (19)	0.0405 (7)	
H5	0.3286	−0.0473	0.1895	0.049*	
C6	0.0921 (3)	0.0255 (3)	0.3116 (2)	0.0409 (7)	
H6	−0.0039	0.0653	0.3468	0.049*	
C7	0.2808 (3)	−0.2882 (3)	0.4521 (2)	0.0424 (7)	
C8	0.5957 (3)	−0.2995 (3)	0.1376 (2)	0.0409 (7)	
C9	0.6438 (4)	0.1960 (4)	0.1234 (2)	0.0602 (9)	
H9C	0.5507	0.2751	0.0956	0.072*	
H9D	0.7375	0.2286	0.1155	0.072*	
C10	0.6107 (5)	0.1792 (5)	0.2242 (3)	0.0809 (13)	
H10A	0.5138	0.1535	0.2327	0.121*	
H10B	0.5952	0.2735	0.2538	0.121*	
H10C	0.7015	0.0997	0.2521	0.121*	
C11	0.9244 (4)	0.1953 (4)	0.9025 (2)	0.0577 (9)	
H11	0.9201	0.1350	0.9557	0.069*	
N6	1.0609 (3)	0.1611 (3)	0.85410 (18)	0.0512 (7)	
C13	1.0645 (4)	0.2479 (3)	0.7803 (2)	0.0457 (7)	
H13	1.1613	0.2244	0.7453	0.055*	
C14	0.9344 (3)	0.3714 (3)	0.7514 (2)	0.0404 (7)	
C15	0.7945 (4)	0.4035 (4)	0.8032 (2)	0.0533 (8)	
H15	0.7026	0.4867	0.7865	0.064*	
C16	0.9506 (3)	0.4637 (3)	0.6721 (2)	0.0419 (7)	
H16	1.0508	0.4406	0.6406	0.050*	
C17	0.7345 (3)	0.7807 (3)	0.53873 (19)	0.0417 (7)	
H17	0.747 (3)	−0.006 (3)	0.099 (2)	0.067 (12)*	

### Atomic displacement parameters (Å^2^)

**Table:**

	*U*^11^	*U*^22^	*U*^33^	*U*^12^	*U*^13^	*U*^23^
Fe1	0.0384 (3)	0.0424 (4)	0.0381 (4)	−0.0086 (3)	0.0034 (2)	0.0106 (3)
N1	0.0387 (13)	0.0410 (14)	0.0383 (13)	−0.0092 (11)	0.0062 (10)	0.0063 (11)
N2	0.0372 (13)	0.0431 (14)	0.0345 (13)	−0.0130 (11)	0.0060 (10)	0.0047 (11)
N3	0.0344 (13)	0.0445 (14)	0.0391 (13)	−0.0085 (11)	0.0080 (10)	0.0102 (11)
N4	0.0418 (15)	0.073 (2)	0.0584 (18)	0.0057 (14)	0.0150 (13)	0.0275 (15)
N5	0.0464 (16)	0.0499 (16)	0.0545 (16)	−0.0076 (12)	0.0044 (12)	0.0220 (14)
C12	0.0473 (19)	0.075 (2)	0.058 (2)	−0.0233 (18)	0.0052 (16)	0.0173 (18)
N7	0.0404 (14)	0.0469 (15)	0.0407 (14)	−0.0173 (12)	−0.0005 (11)	0.0076 (11)
N8	0.0379 (13)	0.0464 (15)	0.0485 (15)	−0.0129 (11)	0.0044 (11)	0.0104 (12)
N9	0.0362 (15)	0.071 (2)	0.0734 (19)	−0.0046 (13)	0.0092 (13)	0.0306 (16)
O1	0.0492 (14)	0.0503 (14)	0.0551 (14)	−0.0065 (11)	−0.0113 (11)	−0.0028 (11)
S1	0.0399 (4)	0.0577 (5)	0.0429 (5)	−0.0078 (4)	0.0058 (3)	0.0164 (4)
S2	0.0511 (5)	0.0507 (5)	0.0736 (6)	−0.0045 (4)	0.0042 (4)	0.0327 (4)
S3	0.0410 (4)	0.0548 (5)	0.0520 (5)	−0.0077 (4)	0.0059 (4)	0.0184 (4)
C1	0.0456 (17)	0.0447 (17)	0.0418 (17)	−0.0112 (14)	0.0055 (13)	0.0094 (14)
C2	0.0442 (17)	0.0364 (16)	0.0482 (18)	0.0007 (13)	0.0010 (14)	0.0061 (14)
C3	0.0349 (15)	0.0430 (17)	0.0404 (16)	−0.0061 (13)	0.0061 (12)	−0.0018 (13)
C4	0.0319 (14)	0.0388 (15)	0.0347 (15)	−0.0115 (12)	0.0024 (11)	−0.0008 (12)
C5	0.0370 (15)	0.0333 (15)	0.0431 (16)	−0.0036 (12)	0.0037 (12)	0.0090 (13)
C6	0.0350 (15)	0.0429 (17)	0.0403 (16)	−0.0090 (13)	0.0086 (12)	0.0013 (13)
C7	0.0378 (16)	0.0458 (17)	0.0401 (16)	−0.0114 (13)	0.0033 (13)	0.0044 (13)
C8	0.0312 (14)	0.0402 (17)	0.0454 (17)	−0.0063 (12)	0.0083 (12)	0.0018 (14)
C9	0.066 (2)	0.062 (2)	0.059 (2)	−0.0292 (19)	−0.0024 (17)	−0.0029 (18)
C10	0.103 (3)	0.092 (3)	0.063 (3)	−0.054 (3)	0.015 (2)	−0.007 (2)
C11	0.060 (2)	0.064 (2)	0.053 (2)	−0.0279 (18)	−0.0082 (17)	0.0184 (17)
N6	0.0501 (16)	0.0493 (16)	0.0521 (16)	−0.0155 (13)	−0.0111 (13)	0.0132 (13)
C13	0.0415 (17)	0.0480 (18)	0.0475 (18)	−0.0159 (14)	−0.0004 (13)	0.0023 (14)
C14	0.0389 (16)	0.0409 (17)	0.0428 (16)	−0.0161 (13)	−0.0033 (13)	0.0042 (13)
C15	0.0388 (17)	0.056 (2)	0.060 (2)	−0.0117 (15)	−0.0008 (15)	0.0154 (16)
C16	0.0389 (16)	0.0433 (17)	0.0438 (17)	−0.0157 (14)	0.0018 (13)	0.0029 (13)
C17	0.0406 (16)	0.0466 (17)	0.0365 (16)	−0.0139 (13)	0.0010 (13)	0.0025 (13)

### Geometric parameters (Å, °)

**Table:**

Fe1—N5^i^	2.140 (3)	S1—C7	1.690 (3)
Fe1—N5	2.140 (3)	S2—C8	1.623 (3)
Fe1—O1^i^	2.196 (2)	S3—C17	1.677 (3)
Fe1—O1	2.196 (2)	C1—C2	1.377 (4)
Fe1—N1	2.339 (2)	C1—H1	0.9500
Fe1—N1^i^	2.339 (2)	C2—C3	1.375 (4)
N1—C5	1.332 (3)	C2—H2	0.9500
N1—C1	1.352 (4)	C3—C4	1.385 (4)
N2—C6	1.280 (4)	C3—H3A	0.9500
N2—N3	1.368 (3)	C4—C5	1.388 (4)
N3—C7	1.350 (4)	C4—C6	1.453 (4)
N3—H3	0.8800	C5—H5	0.9500
N4—C7	1.312 (4)	C6—H6	0.9500
N4—H4A	0.8800	C9—C10	1.498 (5)
N4—H4B	0.8800	C9—H9C	0.9900
N5—C8	1.156 (4)	C9—H9D	0.9900
C12—C11	1.368 (5)	C10—H10A	0.9800
C12—C15	1.377 (4)	C10—H10B	0.9800
C12—H12	0.9500	C10—H10C	0.9800
N7—C16	1.274 (4)	C11—N6	1.332 (4)
N7—N8	1.373 (3)	C11—H11	0.9500
N8—C17	1.361 (4)	N6—C13	1.332 (4)
N8—H8	0.8800	C13—C14	1.395 (4)
N9—C17	1.312 (4)	C13—H13	0.9500
N9—H9A	0.8800	C14—C15	1.383 (4)
N9—H9B	0.8800	C14—C16	1.453 (4)
O1—C9	1.408 (4)	C15—H15	0.9500
O1—H17	0.816 (10)	C16—H16	0.9500
			
N5^i^—Fe1—N5	180.00 (14)	C2—C3—H3A	120.2
N5^i^—Fe1—O1^i^	88.99 (10)	C4—C3—H3A	120.2
N5—Fe1—O1^i^	91.01 (10)	C3—C4—C5	117.5 (2)
N5^i^—Fe1—O1	91.01 (10)	C3—C4—C6	120.6 (2)
N5—Fe1—O1	88.99 (10)	C5—C4—C6	121.9 (3)
O1^i^—Fe1—O1	180.00 (14)	N1—C5—C4	123.9 (3)
N5^i^—Fe1—N1	88.53 (9)	N1—C5—H5	118.0
N5—Fe1—N1	91.47 (9)	C4—C5—H5	118.0
O1^i^—Fe1—N1	85.58 (9)	N2—C6—C4	119.9 (2)
O1—Fe1—N1	94.42 (9)	N2—C6—H6	120.0
N5^i^—Fe1—N1^i^	91.47 (9)	C4—C6—H6	120.0
N5—Fe1—N1^i^	88.53 (9)	N4—C7—N3	116.9 (3)
O1^i^—Fe1—N1^i^	94.42 (9)	N4—C7—S1	123.1 (2)
O1—Fe1—N1^i^	85.58 (9)	N3—C7—S1	119.9 (2)
N1—Fe1—N1^i^	180.00 (12)	N5—C8—S2	179.0 (3)
C5—N1—C1	117.4 (2)	O1—C9—C10	113.3 (3)
C5—N1—Fe1	120.69 (18)	O1—C9—H9C	108.9
C1—N1—Fe1	121.52 (18)	C10—C9—H9C	108.9
C6—N2—N3	116.9 (2)	O1—C9—H9D	108.9
C7—N3—N2	119.2 (2)	C10—C9—H9D	108.9
C7—N3—H3	120.4	H9C—C9—H9D	107.7
N2—N3—H3	120.4	C9—C10—H10A	109.5
C7—N4—H4A	120.0	C9—C10—H10B	109.5
C7—N4—H4B	120.0	H10A—C10—H10B	109.5
H4A—N4—H4B	120.0	C9—C10—H10C	109.5
C8—N5—Fe1	170.5 (3)	H10A—C10—H10C	109.5
C11—C12—C15	119.4 (3)	H10B—C10—H10C	109.5
C11—C12—H12	120.3	N6—C11—C12	122.9 (3)
C15—C12—H12	120.3	N6—C11—H11	118.5
C16—N7—N8	117.2 (2)	C12—C11—H11	118.5
C17—N8—N7	119.6 (2)	C11—N6—C13	117.4 (3)
C17—N8—H8	120.2	N6—C13—C14	123.9 (3)
N7—N8—H8	120.2	N6—C13—H13	118.1
C17—N9—H9A	120.0	C14—C13—H13	118.1
C17—N9—H9B	120.0	C15—C14—C13	117.1 (3)
H9A—N9—H9B	120.0	C15—C14—C16	122.6 (3)
C9—O1—Fe1	126.7 (2)	C13—C14—C16	120.3 (3)
C9—O1—H17	112 (3)	C12—C15—C14	119.2 (3)
Fe1—O1—H17	115 (3)	C12—C15—H15	120.4
N1—C1—C2	122.6 (3)	C14—C15—H15	120.4
N1—C1—H1	118.7	N7—C16—C14	120.5 (3)
C2—C1—H1	118.7	N7—C16—H16	119.7
C3—C2—C1	119.1 (3)	C14—C16—H16	119.7
C3—C2—H2	120.5	N9—C17—N8	116.1 (3)
C1—C2—H2	120.5	N9—C17—S3	123.4 (2)
C2—C3—C4	119.6 (3)	N8—C17—S3	120.5 (2)

Symmetry codes: (i) −*x*+1, −*y*, −*z*.

### Hydrogen-bond geometry (Å, °)

**Table:**

*D*—H···*A*	*D*—H	H···*A*	*D*···*A*	*D*—H···*A*
N9—H9B···S3^ii^	0.88	2.67	3.533 (3)	165
N4—H4B···S1^iii^	0.88	2.52	3.373 (3)	164
N4—H4A···S2	0.88	2.80	3.360 (3)	123
N3—H3···S3^iv^	0.88	2.59	3.414 (3)	156

Symmetry codes: (ii) −*x*+1, −*y*+2, −*z*+1; (iii) −*x*+1, −*y*−1, −*z*+1; (iv) *x*−1, *y*−1, *z*.
